# Cross-Tissue Transcriptomic Analysis Leveraging Machine Learning Approaches Identifies New Biomarkers for Rheumatoid Arthritis

**DOI:** 10.3389/fimmu.2021.638066

**Published:** 2021-06-08

**Authors:** Dmitry Rychkov, Jessica Neely, Tomiko Oskotsky, Steven Yu, Noah Perlmutter, Joanne Nititham, Alexander Carvidi, Melissa Krueger, Andrew Gross, Lindsey A. Criswell, Judith F. Ashouri, Marina Sirota

**Affiliations:** ^1^ Bakar Computational Health Sciences Institute, University of California San Francisco, San Francisco, CA, United States; ^2^ Department of Surgery, University of California San Francisco, San Francisco, CA, United States; ^3^ Department of Pediatrics, University of California San Francisco, San Francisco, CA, United States; ^4^ Rosalind Russell/Ephraim P. Engleman Rheumatology Research Center, Division of Rheumatology, Department of Medicine, University of California San Francisco, San Francisco, CA, United States; ^5^ Howard Hughes Medical Institute, University of California San Francisco, San Francisco, CA, United States; ^6^ Department of Medicine, Oregon Health & Science University, Portland, OR, United States; ^7^ Institute for Human Genetics (IHG), University of California San Francisco, San Francisco, CA, United States; ^8^ Department of Medicine, University of California San Francisco, San Francisco, CA, United States; ^9^ Department of Orofacial Sciences, University of California San Francisco, San Francisco, CA, United States

**Keywords:** rheumatoid arthritis, biomarker, gene expression, machine learning, synovium, blood

## Abstract

There is an urgent need to identify biomarkers for diagnosis and disease activity monitoring in rheumatoid arthritis (RA). We leveraged publicly available microarray gene expression data in the NCBI GEO database for whole blood (N=1,885) and synovial (N=284) tissues from RA patients and healthy controls. We developed a robust machine learning feature selection pipeline with validation on five independent datasets culminating in 13 genes: *TNFAIP6*, *S100A8*, *TNFSF10*, *DRAM1*, *LY96*, *QPCT*, *KYNU*, *ENTPD1*, *CLIC1*, *ATP6V0E1*, *HSP90AB1*, *NCL* and *CIRBP* which define the RA score and demonstrate its clinical utility: the score tracks the disease activity DAS28 (p = 7e-9), distinguishes osteoarthritis (OA) from RA (OR 0.57, p = 8e-10) and polyJIA from healthy controls (OR 1.15, p = 2e-4) and monitors treatment effect in RA (p = 2e-4). Finally, the immunoblotting analysis of six proteins on an independent cohort confirmed two proteins, *TNFAIP6*/TSG6 and *HSP90AB1*/HSP90.

## Introduction

Rheumatoid arthritis (RA) is a systemic inflammatory condition characterized by a symmetric and destructive distal polyarthritis. Undiagnosed and untreated, RA can progress to severe joint damage, involve other organ systems and predispose individuals to cardiovascular disease (1, 2). While our understanding of disease pathogenesis has greatly improved and the number of available, effective therapeutics has significantly increased, there remains significant barriers to caring for patients with RA, and they continue to suffer from the morbidity and mortality associated with the disease. There is an urgent need to develop objective biomarkers for the early diagnosis and prompt initiation of disease-modifying therapy during the so-called “window of opportunity” (3–6). Additionally, clinicians need tests to help accurately assess disease activity or treatment targets in order to adjust therapy appropriately. Identification of biomarkers would greatly add to clinicians’ existing toolset used to evaluate patients with RA, helping to improve outcomes and alleviate the suffering caused by this prevalent disease.

Over the past decade, advances in genomic sequencing technology have greatly contributed to our understanding of inflammatory diseases and informed development of effective therapeutics. Transcriptomics provides a lens into the specific genes over- or under-expressed in a disease yielding insights into cellular responses. Given the numerous transcriptomic datasets that have been generated and made publicly available, there are now opportunities to combine these datasets in a meta-analytic fashion for unbiased computational biomarker discovery. Meta-analysis is a systematic approach to combine and integrate cohorts to study a disease condition which provides enhanced statistical power due to a higher number of samples when combined. Additionally, it provides an opportunity for leveraging all the disease heterogeneity combined from multiple smaller studies across diverse populations creating a more robust signature and better recognition of direct disease drivers as well as disease subtyping and patient stratification. Moreover, integrating datasets generated from the multiple target tissues within a given disease further strengthens the associations identified (7–13). This approach has been successfully applied to the study of antineutrophil cytoplasmic antibody (ANCA)-associated vasculitis (14), dermatomyositis (15) and systemic lupus erythematosus (9). These large datasets also present an opportunity to apply advanced machine learning techniques that were not previously feasible computationally, allowing for interrogation of the data with new and unbiased approaches.

Multiple studies have attempted to identify RA transcriptomic signatures in blood (13, 16–18) and in synovial tissue (19, 20) separately or in cross-tissue analysis (21, 22). The tissue-specific studies have found very few overlapping signals. The integrative meta-analysis studies combined a few datasets from each tissue (21, 22) to identify an overlap of dysregulated genes and to recognize similarities and differences in disease pathways in both tissues. While this type of approach allows better understanding of the disease, a corresponding set of biomarkers is often redundant and requires extensive prioritization analysis and validation. Thus, more rigorous approaches for biomarker search with a built-in prioritization procedure are needed.

In this study, we leveraged publicly available transcriptomic datasets generated from microarray and RNA sequencing (RNA-seq) platforms from over 2,000 samples from whole blood and synovial tissue of patients with RA. After combining these datasets using a well-described meta-analytic pipeline (23) and describing the expression pathways and cell types present in RA tissues, we developed and applied a robust machine learning and feature selection approach to identify unique and independent biomarkers which were subsequently refined and validated on test data. We then evaluated the diagnostic utility of this set of biomarkers and the correlation with disease activity measures to inform future clinical studies. The development of an effective blood test for the diagnosis and monitoring of RA can add valuable information to the physician’s assessment and help inform decision-making to improve the morbidity and quality of life for patients with RA.

## Materials and Methods

### Discovery Data Collection and Processing

**Figure 1:**
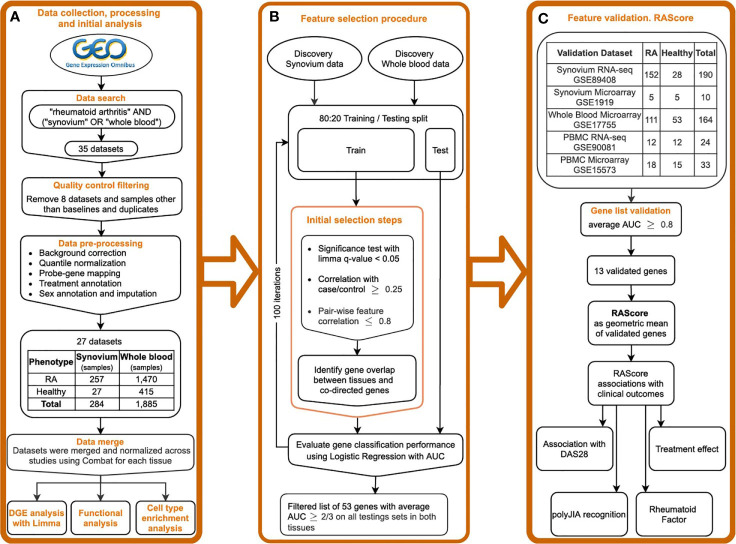
Study overview. **(A)** Public data collection, processing and DGE analysis. **(B)** Feature selection pipeline. **(C)** Gene list validation on the independent datasets. Introducing the RA Score as a geometric mean of validated genes and its association with clinical outcomes.

We carried out a comprehensive search for publicly available microarray data in the NCBI Gene Expression Omnibus (24) (GEO) database (http://www.ncbi.nlm.nih.gov/geo/) for whole blood and synovial tissues in rheumatoid arthritis and healthy controls using the keywords “rheumatoid arthritis”, “synovium”, “synovial”, “biopsy” and “whole blood”, among organisms “Homo Sapiens” and study type “Expression profiling by array” (**Figure 1A**) by March 2019. Datasets were excluded when samples were poorly annotated or run on platforms with small numbers of probes. This search yielded 13 synovial datasets, which included 257 biopsy samples from subjects with RA and 27 from healthy controls obtained during joint or trauma surgeries (**Supplementary Table 1**). We identified 14 whole blood datasets with 1,885 samples: 1,470 RA patients and 415 healthy controls (**Supplementary Table 1**).

Raw data was downloaded and processed using R language version 3.6.5 (25) and the Bioconductor (26) packages *SCAN.UPC* (27), *affy* (28) and *limma* (29). Processing steps included background correction, log2-transformation and intra-study quantile normalization (**Figure 1A**). For the Affymetrix platform, we used the R package *SCAN.UPC* (27) which is a single-array method that normalizes samples independently from others in a dataset and has been shown to be robust to presence of any possible outliers (27). The mapping between probes and Entrez gene IDs was performed using custom CDF files from the BrainArray resource (30) version 22. For the Agilent and Illumina platforms, the non-normalized raw data were downloaded and processed using the *neqc()* function with default parameters within the *limma* R package (29) that utilizes negative control genes or gene detection significance. For datasets with no negative control probes and detection p-values available, the similar processing steps were performed by applying the *backgroundCorrect()* function from the *limma* package using the *mle normexp* method with offset *16* followed by *log2* transformation and quantile normalization. The probe-gene mapping was implemented using the information from the *biomaRt* database (31) or GPL files from GEO. The data merging was followed by normalization across batches using ComBat within the R package *sva* (32). The dimensionality reduction plots before and after normalization are shown in **Supplementary Figure 1**. To perform an additional evaluation of the batch correction on the discovery datasets, we split the corrected and uncorrected data into training and testing sets with a 2:1 ratio and trained a Random Forest classifier on the first 20 principal components to predict a dataset of origin. Multinomial logistic loss (mLogLoss) metric was used to evaluate the prediction performance. The mLogLoss results for prediction of batch corrected synovium data were 2.78 in contrast to 0.37 in uncorrected data, and 1.06 and 0.01 in, respectively, batch corrected and uncorrected blood data. Additionally, we performed a correlation analysis of the first 10 principal components with batch categories (**Supplementary Figure 1I**) using Kruskal-Wallis test. We found no statistical significance in correlation with a batch in synovium and dramatic improvement in blood compared to the original data (**Supplementary Figures 1J, K** **)**.

After merging studies, the total number of common genes was 11,057 in synovium and 14,596 in whole blood. The code for the preprocessing steps is available here: https://github.com/drychkov/RA_biomarkers.

### Validation Data Collection and Processing

Five additional datasets from GEO were identified and downloaded: synovium microarray and RNA-seq, PBMC microarray and RNA-seq and whole blood microarray datasets (**Supplementary Table 1**). Microarray data was processed as described above but separately from the discovery data. RNA-seq data from GSE89408 were downloaded in a form of processed data of feature counts, which were normalized using the variance stabilizing transformation function *vst()* from the R package *DESeq2* (33). RNA-seq data from GSE90081 were downloaded in a processed form of Fragments Per Kilobase Million (FPKM) counts, which were converted to Transcripts Per Kilobase Million (TPM) counts followed by log2 transformation with 0.1 offset.

### Differential Gene Expression and Pathway Analysis

Differentially expressed genes were identified using a linear model from the R package *limma* (29). To account for factors related to gene expression, the imputed sex and treatment categories were used as covariates. Treatment types were categorized based on the drug class (**Supplementary Table 2** **)**. For 877 (40%) samples without sex annotations, sex was imputed using the average expression of Y chromosome genes. Significance for differential expression was defined using the cutoff of FDR p-value < 0.05 and abs(FC) > 1.2. Pathway analysis of differentially expressed genes was performed using the package *clusterProfiler* (34) with the Reactome database. To assess statistical significance of gene overlaps we computed p-values using the hypergeometric test with 10,071 total, background, number of genes.

### Cell Type Enrichment Analysis

In order to estimate the presence of certain cell types in a tissue, we leveraged the cell type enrichment analysis tool, *xCell* (35) which computes enrichment scores for 64 immune and stromal cells based on gene expression data. We limited our analysis to 53 types of stromal, hematopoietic and immune cells we expected to be present in blood and synovium. The cell types with a detection p-value greater than 0.2 taken as a median across all samples in a tissue were filtered. Non-parametric Wilcoxon-Mann-Whitney test with multiple testing correction with Benjamini-Hochberg approach (cut-off 0.05) was used to assess significantly enriched cell types in synovium and whole blood in RA compared to healthy control subjects. The effect size of each cell type was estimated by computing the ratio of the mean enrichment score in RA patients over the mean score in healthy individuals.

### Feature Selection Pipeline

The feature selection procedure was partially described by Perez-Riverol et al. (36) and is represented in **Figure 1B**. First, for each tissue, the data were split into training and testing sets in an 80:20 ratio with random sample selection and class distribution preservation using the function *createDataPartition()* from the R package *caret* (37). Within each training set, a set of significant genes was identified using *limma* FDR p-value < 0.05. Pearson correlation coefficient was computed with the case-control status for each significant gene and those with r < 0.25 were filtered out. For robustness and reducing gene redundancy, we computed gene pair-wise correlations and removed genes with correlation greater than 0.8. Next, we overlapped the gene sets from both tissues and filtered out any genes differentially expressed in opposite directions in synovium and blood. To monitor statistical significance of gene overlaps we computed p-values using the hypergeometric test. To evaluate each gene’s performance in distinguishing RA from healthy samples, we trained a logistic regression model per gene on a training set for each tissue and tested on a testing set using area under receiver operating characteristic (AUROC) curve as a performance measure. By using the AUROC as final metric, we aimed to use a complimentary approach starting with a traditional method of differential expression (limma) which has a strong biological interpretation and then making sure that it is used as a starting point for the predictions that we have *via* the machine learning approach, therefore they are not just based on accuracy or predictive value.

We repeated these steps 100 times to minimize bias of a random split into training and testing sets. From the resulting 100 gene sets, any gene that was found in each set in both tissues was further assessed. The AUC performance of each gene was averaged, and its standard deviation was calculated. We then set the AUC threshold to 2/3 and applied this criterion to the testing results to identify the genes with the best performance, the feature selected (FS) genes.

### Feature Validation and RA Score

To evaluate and confirm superiority of the set of the FS genes over the set of the common DE genes, we trained machine learning models on the discovery blood data with these two gene sets and tested them on five separate independent datasets. As some genes were not present in all sets, the gene sets were reduced to the genes that were found in all five sets. To bring datasets to the same scale, we applied a z-scaling transformation to both discovery and validation datasets. We used three machine learning models: Logistic Regression, Elastic Net and Random Forest, to compare the gene sets performance using AUROC.

Next, to further validate the FS genes identified in our pipeline, we trained a Logistic Regression model for each FS gene individually on the discovery data and tested on the validation sets (**Figure 1C**). Since the primary aim of the study was to identify biomarkers for future clinical tests based on blood, we used the discovery blood data for training the model. AUROC was used as a performance measure. With the goal of choosing the strongest features, the stricter threshold 0.8 of averaged AUC on the validation datasets was chosen for further gene selection. The selected genes were used to create the RA Score, computed by subtracting the geometric mean expression of the down-regulated genes from the geometric mean expression of the up-regulated genes. By creating the RA score based on the computation of geometric means, we were aiming for better interpretability and ease of clinical application of the findings. The thirteen RA Score panel genes were meant to perform independently of each other, therefore the score would still work if any of the genes failed in further validation analysis or were unavailable in a clinical test.

Next, to assess the clinical value of the selected genes and the RA Score, we identified datasets with samples that included values for the disease activity score based on the 28 examined joints (DAS28) (38). We computed the Pearson correlation coefficients of the RA Score and expression levels of the RA Score genes with DAS28. Eight datasets with both RA and Osteoarthritis (OA) samples (**Supplementary Table 1**) were used to evaluate the ability of the RA Score to distinguish RA from OA. To report the summarized statistics as a combined p-value, Fisher’s method implemented in the R package *metap* was applied. The summarized odds ratio was computed by bootstrapping. GSE74143 was used to test the difference in RA Score between RA sub-phenotypes with and without rheumatoid factor by applying Student’s t-test. GSE45876 and GSE93272 were used to test the RA Score difference between treated and untreated RA patients *via* Student’s t-test. Additionally, we leveraged 10 datasets to test the ability of the RA Score to recognize polyarticular juvenile idiopathic arthritis (polyJIA), an inflammatory arthritis similar to RA affecting children under 16 years of age, by calculating the Odds Ratio (**Supplementary Table 1**).

### Immunoblot Analysis

Patients with RA were recruited at University of California San Francisco Rheumatology Clinics. Blood samples and clinical measurements were obtained at the time of enrollment. Clinical measurements included Clinical Disease Activity Index (CDAI), erosion status (presence vs. absence), rheumatoid factor (positive vs. negative) and anti-cyclic citrullinated peptide (anti-CCP) autoantibodies (positive vs. negative). Healthy controls were recruited through local advertising and ResearchMatch (39), a national health volunteer registry that was created by several academic institutions and supported by the U.S. National Institutes of Health as part of the Clinical Translational Science Award (CTSA) program. Controls were matched to RA patients by age, gender and race. Written informed consent was obtained from all participants and Institutional Review Board approval was obtained.

Frozen human PBMCs from 8 patients with RA (87.5% were seropositive) and 7 healthy controls were thawed, washed, resuspended in RPMI media. Those determined to have viability >89% (ViCell Counter) were used to make lysates for western blot. Cells were pelleted and then lysed by directly adding 10% NP-40 lysis buffer to the final concentration of 1% NP40 (containing inhibitors of 2 mM NaVO4, 10 mM NaF, 5 mM EDTA, 2 mM PMSF, 10 μg/ml Aprotinin, 1 μg/ml Pepstatin and 1 μg/ml Leupeptin) as previously described (40). Lysates were placed on ice and centrifuged at 13,000 g to pellet cell debris. Supernatants were mixed with a 6X loading buffer containing BME. Proteins were separated on 10% Bis-Tris gels (Thermo Fisher) and transferred to Immobilon-P polyvinylidene difluoride membranes (Millipore) *via* standard tank transfer techniques. Primary staining was performed with the following antibodies: TSG-6 (Santa Cruz Biotechnology: sc-377277, clone: E-1), Protein S100A8/Calgranulin A (Santa Cruz Biotechnology: sc-48352, clone: C-10), CD39 (MyBiosource: MBS2541905), HSP90beta (Cell Signaling Technology: #5087), Ly96/MD-2 (Novus Biologicals: NB100-56655), TRAIL (Cell Signaling Technology: #3219S). Membranes were blocked using a TBS-T buffer containing 2% BSA and probed with primary antibodies as described, overnight at 4°C. The following day, blots were rinsed and incubated with HRP-conjugated secondary antibodies. Horseradish peroxidase (HRP)-conjugated secondary antibodies from Southern Biotech and blots were visualized with SuperSignal ECL reagent or SuperSignal West Femto maximum sensitivity substrate (Pierce Biotechnology) on Chemi-Doc Image Lab station (Bio-Rad).

Each protein was measured on a set of two immunoblots and normalized to the beta-actin level. In order to combine and normalize measured protein amounts from both blots for further analysis, we applied an empirical Bayes approach *ComBat* implemented in R package *sva* (32). Each of two pairs of control replicates were averaged. The Wilcoxon-Mann-Whitney test was used to compare groups and unadjusted p-values were reported.

## Results

### Cross-Tissue Differential Expression and Pathway Analysis Reveals Significant Similarities on Gene and Pathway Levels

**Figure 2:**
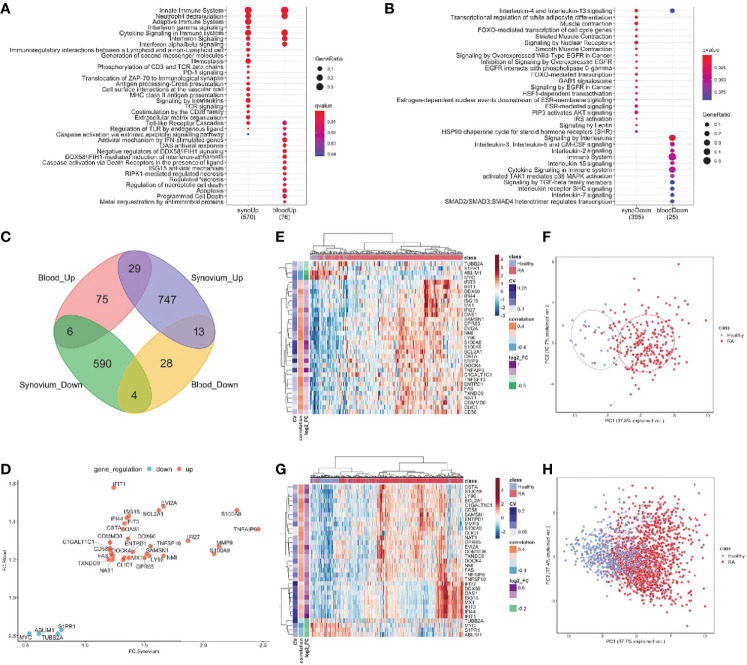
DE genes overlapped between synovium and whole blood tissues. Top Reactome common and different pathways for **(A)** Up-regulated and **(B)** downregulated genes. **(C)** Venn diagram of up- and down-regulated genes in synovium and blood: 29 common up-regulated genes (p =3e-09) and 4 common downregulated genes (p = 0.28). **(D)** Comparison scatter plot of fold changes between common genes in synovium and blood. Heatmap and PCA plots of common genes in **(E, F)** synovium and **(G, H)** blood. Vertical bars in the heatmap plots represent the color-coded coefficients of variation, Pearson correlations and log2 fold changes.

The differential gene expression analysis identified 1,389 genes with 789 up-and 600 down-regulated genes in the synovium (**Supplementary Figures 2A, B** and **Supplementary Table 3**) and 155 genes with 110 up- regulated and 45 down-regulated genes in the blood (**Supplementary Figures 3A, B** and **Supplementary Table 4**). Out of 1,389 genes in synovium, there were 77 up- and 35 down-regulated genes not shared with the blood data. Similarly, out of 155 genes in blood, there were 20 up- and 5 down-regulated genes not shared with the synovium data. The pathway analysis revealed that in both tissues, up-regulated genes shared enrichments in innate immune system, neutrophil degranulation, interferon signaling, cytokine signaling, toll-like receptor (TLR) cascades, regulation of TLR by endogenous ligand and caspase activation *via* extrinsic apoptotic signaling pathways (**Figure 2A**, **Supplementary Figures 2D, E**, **3D, E** and **Supplementary Tables 5**, **6**). However, interferon gamma signaling, immunoregulatory interactions between a lymphoid and non-lymphoid cell, PD-1 signaling were specific for synovium (**Supplementary Table 5**), whereas apoptosis, programmed cell death, antiviral mechanisms, caspase activation *via* death receptors in the presence of ligand were specific for blood (**Supplementary Table 6**). The down-regulated genes were commonly involved only in the interleukin-4 and interleukin-13 signaling pathways (**Figure 2B** and **Supplementary Figures 2F, G**, **3F, G**). Some of the pathways were not shared, suggesting the existence of distinct underlying molecular mechanisms operating in tissues. For example, signaling by interleukins, TCR signaling and MHC class II antigen presentation pathways were specific only for synovium (**Supplementary Tables 5**, **6**). The latter was fully consistent with our previous work demonstrating enrichment of Nur77 – a specific marker of TCR signaling – in joint infiltrating CD4+ T-cells, suggesting that CD4+ T-cells are recognizing intra-articular antigen (40).

When evaluating the overlap between differentially expressed genes in synovium and blood, there were 29 genes commonly up-regulated: *TNFAIP6, S100A8, MMP9, S100A9, IFI27, EVI2A, NMI, BCL2A1, TNFSF10, LY96, SAMSN1, GPR65, DDX60, ISG15, MX1, OAS1, IFI44, ENTPD1, IFIT3, CSTA, CLIC1, IFIT1, DOCK4, NAT1, FAS, C1GALT1C1, CD58, COMMD8, TXNDC9*; and 4 down-regulated genes: *S1PR1, TUBB2A, ABLIM1* and *MYC* (**Figure 2C** and **Supplementary Table 7**, hypergeometric test p = 3e-9). However, the overlap of down-regulated genes did not meet statistical significance (hypergeometric test p = 0.28, **Figure 2D**). The common differentially expressed (DE) genes formed more distinct clusters of RA and control samples for both synovium (**Figures 2E, F** **)** and blood (**Figures 2G, H** **)** than all DE genes for these tissues (**Supplementary Figures 2A, B**, **3A, B** **)**. To test this, we applied an unsupervised k-means classifier on the first two principal components computed from all DE, 33 overlapping and 33 random genes in both synovium and blood data. We evaluated the predicted clusters using a few metrics for classification. In synovium, the clusters were identified for DE genes with Sens = 1, Spec = 0.78 and Prec = 0.31, Recall = 1 (**Supplementary Figure 2B**); 33 overlapping genes with Sens = 1, Spec = 0.79 and Prec = 0.34, Recall = 1 (**Figure 2F**); and 33 random genes with Sens = 0.96, Spec = 0.51 and Prec = 0.17, Recall = 0.96 (**Supplementary Figure 2C**). In blood, the clusters were identified for DE genes with Sens = 0.88, Spec = 0.71 and Prec = 0.46, Recall = 0.88 (**Supplementary Figure 3B**); 33 overlapping genes with Sens = 0.9, Spec = 0.67 and Prec = 0.44, Recall = 0.9 (**Figure 2H**); and 33 random genes with Sens = 0.65, Spec = 0.61 and Prec = 0.32, Recall = 0.65 (**Supplementary Figure 3C**).

The enriched Reactome pathways of these common up-regulated genes included interferon signaling, neutrophil degranulation, regulation of TLR by endogenous ligand and caspase activation *via* extrinsic apoptotic signaling pathway and *via* death receptors in the presence of ligand, whereas down-regulated genes are associated with Interleukin-4 and 13 signaling and cell cycle pathways. These results were consistent with the pathway analysis above.

### Cell-Type Deconvolution Analysis Identifies a Reverse Signal in Blood and Synovium

**Figure 3:**
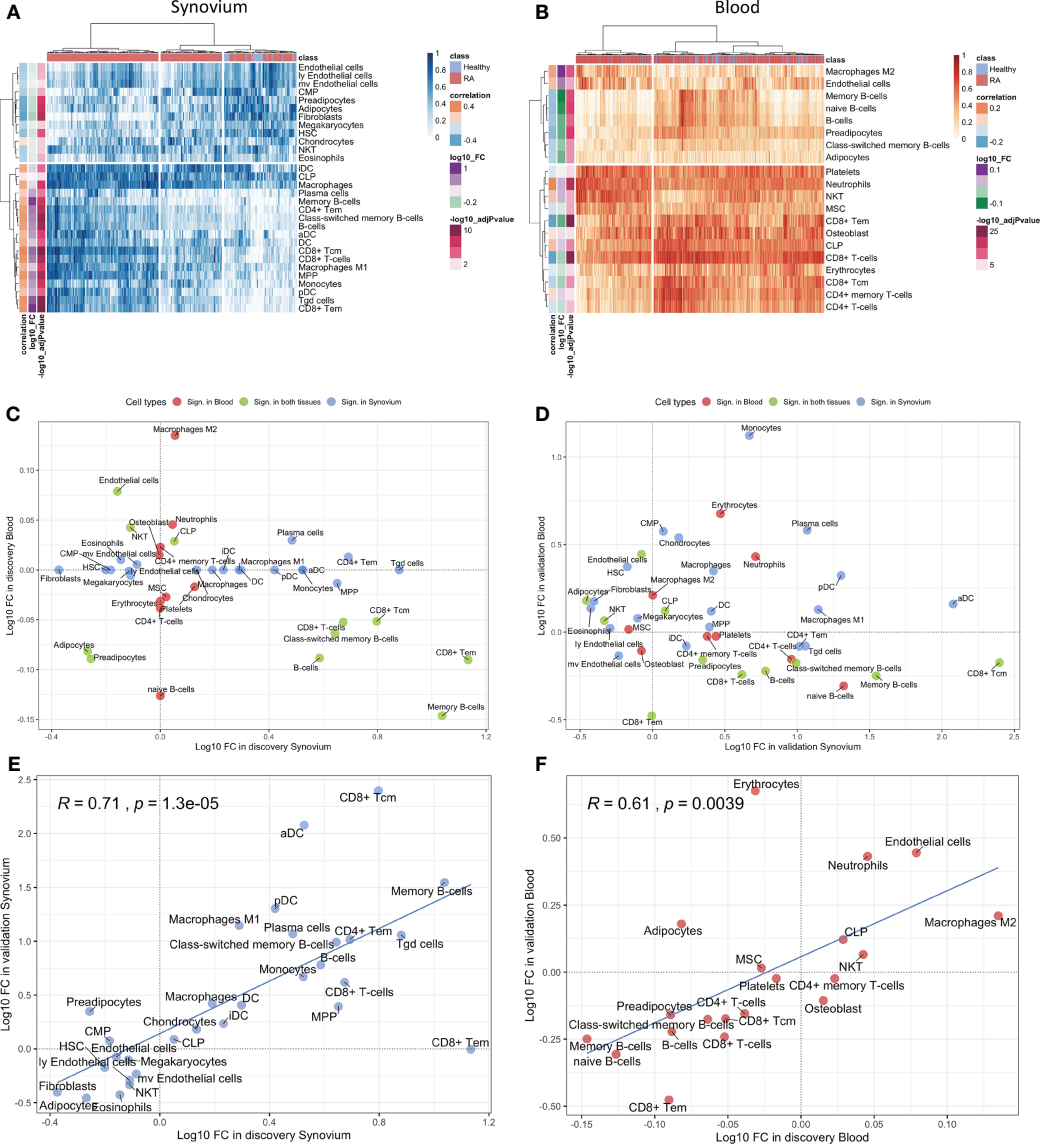
Cell type enrichment analysis for synovium and whole blood tissues. 30 cell types were significant (BH adj p-values < 0.05) in synovium and 20 were significant in whole blood with 11 common cell types between the tisuues. Heatmap plots of significantly enriched cell types in **(A)** synovium and **(B)** blood. The scatter plots comparing log10 transformed fold changes of significant cell types between synovium and blood in **(C)** discovery and **(D)** validation cohorts. The scatter plots comparing log10 transformed fold changes of significant cell types between discovery and validation cohorts in **(E)** synovium and **(F)** blood tissues, with Pearson correlation coefficient and it's p-value.

The cell type enrichment analysis with xCell in synovium revealed the significant enrichment of immune cell types, including, CD4+ and CD8+ T-cells, B-cells, macrophages and dendritic cells in RA samples (**Figure 3A**). However, opposite but weaker associations were seen in whole blood samples with enrichment of T- and B-cells in healthy controls (**Figure 3B**). Lymphocytes, including CD8+ T cells and B cells, were significantly enriched in both tissues, however, these were enriched in opposite directions with enrichment in cases in synovium but enrichment in controls in blood (**Figure 3C**). This finding was confirmed in validation datasets (**Figure 3D**). The significant cell types in synovium and blood showed high correlations in validation data: r = 0.71 (p = 1.3e-5) for synovium (**Figure 3E**) and r = 0.61 (p = 0.004) in blood (**Figure 3F**).

### Machine Learning Feature Selection Strategy to Identify Robust Cross-Tissue Biomarkers of RA

**Figure 4:**
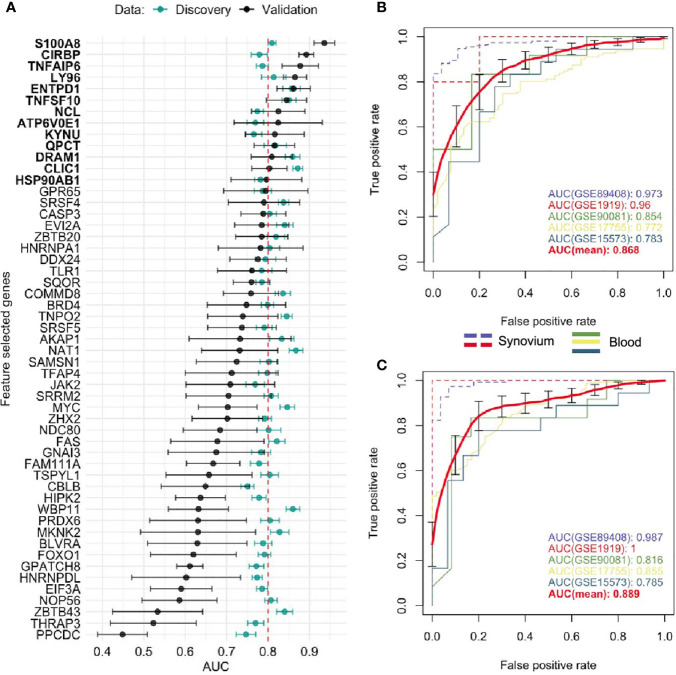
Feature selected genes. **(A)** Mean AUC performance with standard error for each feature selected gene on testing synovium and blood datasets (green) and on five independent validation datasets (black). 13 genes with AUC greater than 0.8 on validation datasets were chosen as the best performing genes. Mean AUC performance with standard errors of a RF model trained on discovery blood data with **(B)** common DE genes and **(C)** feature selected genes on five independent validation datasets. The discovery-based classifiers were held fixed and used once on each validation dataset.

Aiming to determine a more robust list of putative biomarkers that are strongly associated with RA in both synovium and whole blood tissues and have higher predictive power, we applied an iterative feature selection procedure leveraging the gene expression data from both tissues. In the pipeline, only 10,071 genes that were common between synovium and whole blood data were used. At each iteration, only genes found significantly dysregulated in both tissues following the condition of co-directionality were kept (p = 6.3e-10). As a result of these filtering steps, on average 65 up-regulated and 71 down-regulated were selected from each iteration (see *Methods*). From 100 iterations, any gene significantly dysregulated in all the iterations was selected, resulting in a set of 53 genes: 25 upregulated and 28 down-regulated (**Supplementary Table 8**). A summary of the average AUC performance from the 100 iterations for each gene are shown in the **Figure 4A** and **Supplementary Table 8**. The AUC for selected genes in synovial tissue varied with mean 0.853 ± 0.005 for cross-validation in training and 0.866 ± 0.006 for testing sets of the discovery data, whereas for the whole blood the mean AUC was 0.744 ± 0.006 for training and 0.747 ± 0.006 for testing sets. To address the class imbalance and confirm the robustness of using the AUC metric for the feature selection, we additionally computed the area under the precision-recall curve (AUCPR) for each of 53 genes and compared them to AUROC using the Pearson correlation (**Supplementary Figure 4**). We found that the AUCPR was significantly correlated with the AUROC: r = 0.62 (p = 7.7e-7) and r = 0.88 (p = 1.5e-15) for synovium and blood, respectively. Therefore, even though using AUCPR thresholding slight variations in the set of feature selected genes are possible, the gene set based on AUC is still robust.

We leveraged 5 publicly available independent datasets on synovium and blood to validate these results (see *Methods*) (**Supplementary Table 1**). First, we compared the classification performance of the set of 53 feature selected genes to the set of 33 common DE genes (*Methods*). Since not all genes were measured across the validation studies, the sets were reduced to 26 of 33 common DE genes and 38 of 53 feature selected genes. We found the set of 38 feature selected genes has superior performance over the set of 26 common DE genes for all three ML methods (**Supplementary Figure 5**). The largest difference in performance was for the Random Forest model: the model with the common DE genes had an AUC of 0.868 ± 0.043 (95% CI [0.785, 0.951]) (**Figure 4B**), while the model with the feature selected genes performed with 0.889 ± 0.044 (95% CI [0.811, 0.966]) (**Figure 4C**). The Random Forest model trained on the 300 random genes performed with AUC 0.837 ± 0.037 on the validation datasets (**Supplementary Figure 5**).

Next, by using the original threshold of averaged AUC > 2/3 on the validation datasets, 38 (72%) out of 53 genes were marked as validated and by applying the stricter threshold AUC > 0.8 thirteen genes (25%) were finally selected: 10 up-regulated *TNFAIP6, S100A8, TNFSF10, DRAM1, LY96, QPCT, KYNU, ENTPD1, CLIC1* and *ATP6V0E1*, and three down-regulated *HSP90AB1, NCL* and *CIRBP* genes (**Figure 4A** and **Supplementary Figure 6**). Five of the related proteins, TSG-6, MRP-8/Calgranulin-A, TNFSF10/TRAIL, Ly-96 and QC, are known to be normally secreted into blood, while Kynureninase (**Table 1**), HSP 90-beta and CLIC1 are localized to cytosol (41).

**Table 1:** Summary of 13 validated RA Score Panel genes.

Gene	Gene name	Regulation	Discovery Synovium			Discovery Blood			Validation	Protein Secretion
			FC (FDR adj. p-value)	ρ (BH adj. p-value)	AUC	FC (FDR adj. p-value)	ρ (BH adj. p-value)	AUC	AUC	
TNFAIP6	TNF Alpha Induced Protein 6	up	2.46 (4E-06)	0.39 (7E-11)	0.81	1.36 (8E-16)	0.39 (3E-67)	0.77	0.88	Secreted in blood
S100A8	S100 Calcium Binding Protein A8	up	2.28 (7E-05)	0.34 (1E-08)	0.81	1.46 (7E-32)	0.48 (9E-108)	0.81	0.94	Secreted in blood
DRAM1	DNA Damage Regulated Autophagy Modulator 1	up	1.55 (6E-07)	0.46 (3E-15)	0.93	1.18 (8E-15)	0.41 (6E-76)	0.79	0.81	
TNFSF10	TNF Superfamily Member 10	up	1.55 (1E-09)	0.52 (3E-19)	0.9	1.27 (1E-23)	0.44 (4E-88)	0.8	0.84	Secreted in blood
LY96	Lymphocyte Antigen 96	up	1.54 (1E-09)	0.51 (2E-18)	0.94	1.22 (7E-11)	0.28 (2E-35)	0.69	0.87	Secreted in blood
QPCT	Glutaminyl-Peptide Cyclotransferase	up	1.46 (4E-05)	0.39 (7E-11)	0.92	1.19 (4E-10)	0.29 (1E-37)	0.71	0.82	Secreted in blood
KYNU	Kynureninase	up	1.41 (5E-05)	0.36 (1E-09)	0.84	1.17 (2E-11)	0.28 (3E-34)	0.69	0.82	Intracellular or membrane-bound
ENTPD1	Ectonucleoside Triphosphate Diphosphohydrolase 1	up	1.33 (1E-08)	0.52 (2E-19)	0.94	1.21 (2E-16)	0.4 (5E-71)	0.78	0.86	
CLIC1	Chloride Intracellular Channel 1	up	1.32 (5E-08)	0.47 (7E-16)	0.91	1.2 (5E-27)	0.47 (4E-103)	0.84	0.8	Intracellular or membrane-bound
ATP6V0E1	ATPase H+ Transporting V0 Subunit E1	up	1.23 (3E-04)	0.37 (8E-10)	0.84	1.08 (4E-10)	0.28 (3E-35)	0.7	0.82	
NCL	Nucleolin	down	0.83 (2E-05)	-0.39 (4E-11)	0.82	0.88 (4E-09)	-0.32 (2E-44)	0.72	0.82	
CIRBP	Cold Inducible RNA Binding Protein	down	0.8 (3E-05)	-0.41 (4E-12)	0.83	0.91 (2E-10)	-0.33 (2E-47)	0.74	0.89	
HSP90AB1	Heat Shock Protein 90 Alpha Family Class B Member 1	down	0.79 (2E-04)	-0.37 (3E-10)	0.82	0.84 (4E-12)	-0.36 (7E-56)	0.73	0.8	Intracellular or membrane-bound

### Clinical Implications of Transcription Based Disease Score

In order to assess the clinical utility of the 13 validated genes, we introduced a scoring function, RA Score, which is derived by subtracting the geometric mean of expression values of down-regulated genes from the geometric mean of up-regulated genes. With this definition, the RA Score is 2-fold (95% CI [1.8, 2.2], p = 3e-15) larger for RA in comparison to healthy samples in synovium. In whole blood, the RA Score has a mean effect size of 1.37 (95% CI [1.34, 1.4], p = 1e-108). In validation datasets, the RA Score had a mean effect size of 5.5 in synovium (95% CI [3.8, 8.2], p = 1e-10) and 2.4 in blood (95% CI [2.1, 2.8], p = 3e-23). This score showed utility in monitoring disease activity, diagnostics and treatment response and also was generalizable to both RF-positive and RF-negative RA as well as polyJIA.

Four datasets with 411 samples included in our meta-analysis had available disease activity score (DAS28) annotations. Assessing the correlation with DAS28 for each gene individually, the most positively correlated gene was *S100A8* with mean R = 0.28 (95% CI [0.19, 0.37]) and most anti-correlated gene *HSP90AB1* with mean r = -0.23 (95% CI [-0.32, -0.14]) (**Supplementary Figures 7**, **8A** **).** The RA score performed better than any single gene, positively correlated with DAS28 where the average correlation was 0.33 with 95% CI [0.24, 0.41] (**Supplementary Figures 8B, C** **)**, suggesting this score could be helpful as a disease activity biomarker. We also determined the correlation of the RA Score with DAS28 in these datasets separately and obtained Pearson correlation coefficients from 0.25 to 0.43 in blood and 0.31 in synovium (**Supplementary Figure 8B**). Additionally, we tested how the correlation of DAS28 with the RA Score is different from the correlation with the score composed from a random set of 13 genes (**Supplementary Figure 8D**). From 100 iterations the mean Pearson correlation was 0.22 which was significantly different from the correlation with the RA Score 0.33 with p-value = 6e-50 using the Student’s t-test.

**Figure 5:**
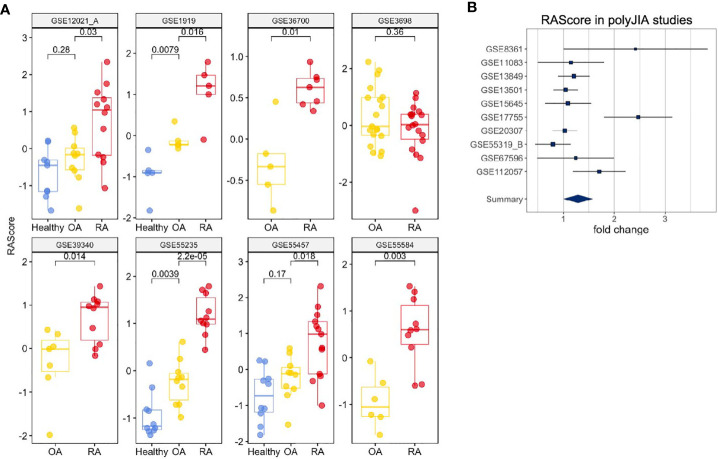
Clinical Interpretation of the RA Score. **(A)** The RA Score distinguishes Healthy, OA and RA samples in synovium. **(B)** The RA Score distinguishes Healthy and polyJIA samples. The p-values were obtained using Student’s t-test.

To investigate the ability of the RA Score to differentiate RA from osteoarthritis (OA), we identified eight datasets that had both RA and OA samples available. **Figure 5A** shows the distributions of RA Score for RA, OA and healthy samples in eight available datasets. In most datasets, the RA Score was able to significantly differentiate OA from RA (OR 0.57, 95% CI [0.34, 0.80], p = 8e-10) and healthy samples (OR 1.53, 95%CI [1.37, 1.69], p = 7.5e-4) suggesting that this score could be useful diagnostically.

One dataset in whole blood, GSE74143, had annotations for RF-positivity. The RA Score performed similarly in both RF-positive and RF-negative RA samples suggesting the applications of this score are generalizable to these RA subtypes (t-test, p = 0.9) (**Supplementary Figure 8E**). Furthermore, we tested the utility of this score in 10 datasets from polyJIA given that this subtype of JIA is most similar to RA and also found comparable performance in the ability to differentiate polyJIA from healthy controls (OR 1.15, 95% CI [1.01, 1.3], p = 2e-4) (**Figure 5B**). Thus, this score could also be useful in the pediatric arthritis population.

Lastly, we observed that the RA Score might also track treatment response. In two synovium and blood datasets, RA patients had transcriptional measurements before and after treatment with disease-modifying antirheumatic drugs (DMARD): methotrexate, tocilizumab (GSE45867, GSE93272) and infliximab (GSE93272). The RA score significantly (p = 2e-4) decreases between pre- and post-treatment measurements (**Supplementary Figure 8F**).

### Western Blot Validation

**Figure 6:**
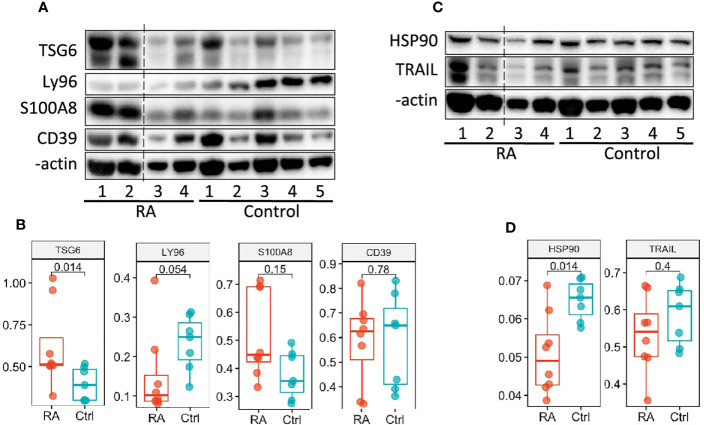
Validation of the RA Score proteins. **(A, C)** Immunoblot analysis of 6 RA Score proteins in unstimulated PBMC lysates from subjects with RA (n=4) and healthy controls (n=5). Data representative of 2 immunoblots. **(B, D)** Box plots are quantification of RA Score protein levels normalized to GAPDH pooled from 2 immunoblot experiments [as shown in **(A, C)** and **Supplemental Figure 8**]; RA (n=8) and healthy control (n=7) samples. Significance determined by Mann-Whitney-Wilcoxon test, **(B, D)**. Dotted line represents lanes removed from non-RA subjects, otherwise immunoblots a and d are montages of the same western blot.

We next examined whether we could validate differences in transcript expression by protein in patients with newly diagnosed RA (validation cohort) prior to treatment initiation based on our RA Score results. Six candidate proteins, for which commercial antibodies are available, were selected by highest absolute fold changes based on transcript expression visible in PBMC’s, as well as in synovial tissue, from our discovery data. Protein levels were assayed using mixed PBMC lysates from the validation cohort (**Figure 6** and **Supplementary Figure 9**). Notably, the TSG6 protein was significantly upregulated in RA PBMCs (**Figure 6B**), while HSP90 was significantly downregulated in the RA validation cohort compared to controls (**Figure 6D**), supporting findings from the transcriptional discovery data. Similar to our RA Score findings, S100A8 was upregulated in some RA samples, though it did not reach statistical significance. Notably, Ly96 protein levels trended in the opposite direction compared to the transcriptional dataset (**Figure 6B**).

## Discussion

In this study, we leveraged publicly available microarray gene expression data from both synovium and peripheral blood tissues in search of putative biomarkers for RA. The cell type enrichment analysis revealed the prevalence of lymphocytes in RA synovial tissue, in contrast to RA blood, likely due to synovial tissue infiltration of immune cells as well as their homing to lymph organs (lymph nodes and spleen) from blood. While lymphopenia, i.e. low concentration of lymphocytes in blood, and synovial infiltration by lymphocytes have been recognized features of RA, the reasons are still not fully understood (42–45). This observation additionally supported our study objective of leveraging the data from both synovium and blood to identify genes expressed concurrently in both tissues. We first applied a conventional approach (13, 46–49) of intersecting the differentially expressed genes from both tissues and obtained a list of 33 common genes. Some of our results showed agreement with previous studies, identifying similar biological processes (e.g., cytokine signaling, immune and defense response, response to biotic stimulus) (13, 18, 21). Furthermore, the differentially expressed genes common to both tissues better distinguished RA cases from healthy controls than all differentially expressed genes combined. While this list of overlapping genes provides valuable insight into disease biology, the predictive ability can be further improved by applying more advanced machine learning methods to prioritize candidate markers and remove redundancy.

Our specific machine learning method identified a robust and non-redundant set of biomarkers concurrently expressed in both RA target tissues. This resulted in 53 protein-coding genes that outperformed the set of the common DE genes in outcome prediction tasks using independent data. Even though the feature selected genes perform better, the common genes still have predictive value which is important to recognize. In further validation steps, we identified and selected 10 up- and three down-regulated genes with the highest performance. The up-regulated genes are highly expressed in diseased synovial tissue, and their elevated protein levels in blood may represent RA biomarkers. However, the combination of these 13 validated genes into a transcriptional gene score, the RA Score, performed better in clinical applications than any one gene and could potentially serve as a clinical blood test for accurate disease diagnosis and monitoring disease activity and response to treatment. Furthermore, this score performed similarly in RF-positive and RF-negative RA and also distinguished polyJIA from healthy controls, broadening the scope of possible clinical applications. Some genes/proteins, e.g. *S100A8*/MRP8, *ENTPD1*/CD39, *KYNU* and *TNFAIP6*, from the score were previously found to be associated with JIA (50–57). Treatment effect was also captured with a significantly lower RA Score for DMARD-treated patients compared to untreated patients. Moreover, since the genes were identified from both blood and synovium and followed the condition of co-directionality, i.e., upregulated or downregulated in both tissues, the RA Score test based on blood only becomes tenable.

The 13 genes identified using these machine learning methods in the feature selection pipeline represent candidate biomarkers in RA. Six of the 13 RA Score Panel genes (*TNFAIP6*, *S100A8*, *TNFSF10*, *LY96*, *ENTPD1* and *CLIC1*) were also among the 33 common DE genes, whereas seven of the 13 RA Score Panel genes (*DRAM1*, *QPCT*, *KYNU*, *ATP6V0E1*, *NCL*, *CIRBP* and *HSP90AB1*) were not. Many of these genes have been described in the literature and studied in the context of RA demonstrating biologic plausibility of the RA Score.

***TNFAIP6***, also known as TSG-6, encodes a secretory protein that is produced in response to inflammatory mediators, with high levels detected in the synovial fluid of patients with RA and OA (58). TNFAIP6 is thought to play an anti-inflammatory role in arthritis and protect destruction of joint cartilage, which has been demonstrated in many arthritis mice models (59). ***S100A8*** is a calcium binding protein that forms a heterodimer with *S100A9* known as calprotectin (*S100A8*/*A9*). Calprotectin is constitutively expressed in neutrophils and monocytes but massively upregulated during inflammatory responses as an important mediator of inflammation (60). Thus, it has been extensively studied as a potential biomarker in several inflammatory diseases including RA. A study investigating the use of calprotectin as measured in sera of RA patients with moderate to severe disease found an association with disease activity, though it was less useful in monitoring radiographic disease progression or treatment response (61). Of note, while the 33 common DE genes set includes *S100A9*, this gene is not one of the 53 FS genes or subsequent 13 RA Score Panel genes likely because our process excludes genes that are highly correlated (pairwise feature correlation greater than 0.8). ***TNFSF10***, also known as TNF-Related Apoptosis Inducing Ligand (TRAIL), encodes a protein that induces apoptosis of tumor cells but is also of interest in RA as it has been thought that *TNFSF10* might induce apoptosis of hyperplastic synoviocytes and reduce immune cell hyperactivity thus providing a protective effect to the joint. However, this is still controversial as there is also evidence that *TNFSF10* may promote joint destruction and exacerbate RA (62). ***LY96***, also known as MD2, encodes a protein which often is a coreceptor with TLR4 forming the TLR4/MD2 complex (63) and has previously been found to be upregulated in patients with rheumatoid arthritis (64–66). ***ENTPD1***, also known as *CD39*, is a gene found to be an expression quantitative trait locus associated in RA affecting levels of CD39+, CD4+ regulatory T-cells (67). In RA, low levels of CD39+ expressing Tregs were associated with methotrexate resistance suggesting this could be a biomarker to predict responders and non-responders (68–70).

Seven of the 13 RA Score Panel genes (*DRAM1*, *QPCT*, *KYNU*, *ATP6V0E1*, *NCL*, *CIRBP* and *HSP90AB1*) were uniquely identified by our machine learning pipeline and not identified by the traditional DE gene overlap method. Four of these genes, *KYNU*, *QPCT*, *CIRBP* and *HSP90AB1* have previously been associated with RA. ***KYNU*** encodes the Kynureninase enzyme which is involved in a pathway of tryptophan metabolism related to immunomodulation and inflammation (71, 72). *KYNU* expression has been found to be increased in chondrocytes and synovial tissue of RA patients compared to healthy patients (73). Moreover, increased tryptophan degradation has been observed in the blood of RA patients (74). The gene ***QPCT*** encodes Glutaminyl-Peptide Cyclotransferase, or QC for short (75), which plays a role in maintaining inflammation (76). A clinical study found that expression of QPCT was significantly increased in the blood and in the fluid from gingival crevices of RA patients compared to healthy controls (77). ***CIRBP*** encodes cold-inducible RNA-binding protein which can be induced under stress and have a cytoprotective role but has also been increasingly recognized for participating in proinflammatory response (78). CIRBP binds to the TLR4/MD2 complex of macrophages and monocytes in the circulation or tissues, thereby activating the NF‐Kappa B pathway and resulting in the release of inflammatory mediators (78, 79). A clinical study measured *CIRBP* mRNA expression of CD14+ monocytes of five healthy and five RA patients and found the relative expression of *CIRBP* was higher in RA patients (80), whereas our analysis found down-regulated *CIRBP* expression in whole blood from RA compared to healthy control. Further study of *CIRBP* in RA patients, particularly single cell analysis, is warranted. ***HSP90AB1*** encodes the protein HSP90β (81). Post-translationally citrullinated isoforms of heat shock protein 90, including citHSP90β, have been identified as potential autoantigens in patients with RA-associated interstitial lung disease (82, 83). We did not find evidence of an association between ***DRAM1***, ***ATP6V0E1*** and ***NCL***, however, these may represent novel genes that warrant future study. *DRAM1* and *NCL* have been shown to be associated with other autoimmune diseases, such as Systemic Lupus Erythematosus (84, 85) and Multiple Sclerosis (86). Furthermore, *DRAM1* is a gene involved in autophagy, an important mechanism for regulating the immune response and autophagy modulation has been postulated as a potential therapeutic in RA (87).

We examined six proteins from RA Score using the immunoblotting technique and confirmed two of them, *TNFAIP6*/TSG6 and *HSP90AB1*/HSP90, with *S100A8*/MRP8 protein trending near significance. Our findings for these proteins highlight that the generation of the RA Score by which to help diagnose patients from RA could be helpful in clinical practice. Further validation studies are underway to examine the transcriptional profile of RA PBMCs on a single cell level, in which transcript changes can be assessed based on cell type, followed by protein validation studies in more RA subjects looking at lysates sorted from specific immune cell subtypes identified by single cell analysis. This will allow us to further fine tune the RA Score genes identified by our analysis. Indeed, studies examining the relationship between protein and mRNA levels highlight the complexity of gene expression regulation (88).

Several limitations of this study should be recognized. A few datasets, especially in whole blood, had significantly more cases than controls with some datasets containing no healthy controls. Therefore, a more robust leave-dataset-out cross validation method, such as fSVA, was not possible to implement in the current feature selection pipeline, though it could be used in the future improvements of our approach with newly generated and better-balanced datasets. The significant class imbalance might result in class variance imbalance and lead to increased type 1 and type 2 errors. To at least partially address this limitation, we included two datasets of healthy individuals to enrich the blood data with control samples. Likewise, the validation cohorts also had an imbalance of cases and controls and two out of three were from PBMC rather than whole blood. The latter was also the case for the validation cohort in the immunoblotting analysis. This could possibly lead to lower AUC performance for genes in the validation datasets but likely does not overestimate the performance of our genes. Class imbalance in datasets also limits the application of the batch correction method ComBat, which was used in this study, as explained by Nygaard et al. (89). In our previous work, we compared ComBat’s performance with other batch correction methods including Remove Unwanted Variation (RUV) (90) using the Guided Principal Component Analysis (91) for batch presence evaluation. We found that ComBat was the most appropriate for both synovium and blood datasets based on the test statistic, δ, which quantifies the proportion of variance owing to batch effects, where its p-value determines whether δ is significantly larger than would be obtained by chance.

Additionally, not all samples were annotated for important covariates such as sex and medication use. All sample annotations were kept from the original publications, though for 40% of samples the sex annotations were not available and they were imputed based on the expression levels of Y chromosome genes. Likewise, most case samples were from RA patients who were taking various medications. Even though the treatments were used in the differential expression analysis as covariates (including untreated patients) there still exists the possibility of confounding.

In this study, we present a robust machine learning pipeline to search for putative biomarkers: each gene went individually through a feature selection procedure with multiple iterations on the discovery data and was independently tested on the validation cohorts. The gene redundancy was decreased selecting the best performing genes in RA association prediction. We apply the pipeline to a set of over 2000 samples to identify the RA Score as a potential diagnostic panel and show some clinical utility. The strength of the RA Score is in the independence of its constituent genes. Further development of the RA Score as a clinical tool requires greater understanding and validation of its component genes with experimental analysis of the protein levels in RA patients and healthy individuals through prospective trials. It is also important to note that the DAS28 score, which was used to evaluate utility, might not be independent of RA phenotype, which potentially can create bias in findings. However, DAS28 is one of the measures that accounts for the disease severity, and its significant correlation of the RA Score might still indicate the potential clinical utility of the latter. An independent longitudinal study would bring better understanding of the early diagnostic and disease monitoring capability of the proposed panel. Additional experiments leveraging single cell technologies will further enhance our understanding of the biology and cell type specific effects in RA allowing us to refine the proposed potential diagnostic strategies.

## Data Availability Statement

The original contributions presented in the study are included in the article/**Supplementary Material**. Further inquiries can be directed to the corresponding author.

## Ethics Statement

The studies involving human participants were reviewed and approved by Institutional Review Board, University of California, San Francisco. The patients/participants provided their written informed consent to participate in this study.

## Author Contributions

MS and DR conceived the study design and analysis plan. DR performed the computational analysis and generated all figures and tables. DR, JNe, TO, and MS performed the interpretation of the data. J Ni, AC, MK, AG, and LC collected clinical materials. JA, SY, and NP preformed the immunoblotting analysis and interpretation. DR, TO, JNe, and MS drafted the manuscript with contributions from JNi and LC. All authors reviewed and edited the manuscript. MS supervised the work. All authors contributed to the article and approved the submitted version.

## Funding

This work was in part supported by Pfizer ASPIRE Grant WI215080, NIH Award P30 AR070155 and the Rheumatology Research Foundation. JA was supported by Rosalind Russell Medical Research Foundation Bechtel Award, the Arthritis National Research Foundation Award, UCSF Center of Rheumatic Diseases, UCSF Research Evaluation & Allocation Committee funded by Esther Memorial fund and K08 AR072144.

## Conflict of Interest

The authors declare that the research was conducted in the absence of any commercial or financial relationships that could be construed as a potential conflict of interest.

## Supplementary Material

Supplementary Table 1 Overview of the Discovery and Validation Studies.

Supplementary Table 2 Treatment Categories.

Supplementary Table 3 Significantly Differentially Expressed Genes – Synovium.

Supplementary Table 4 Significantly Differentially Expressed Genes – Blood.

Supplementary Table 5 Significant Pathways – Synovium.

Supplementary Table 6 Significant Pathways – Blood.

Supplementary Table 7 33 Common DE Genes.

Supplementary Table 8 53 Feature Selected Genes.
