# Supplementary material for: Cross-Tissue Transcriptomic Analysis Leveraging Machine Learning Approaches Identifies New Biomarkers for Rheumatoid Arthritis
Source: Front Immunol. 2021 Jun 8;12:638066. doi: 10.3389/fimmu.2021.638066 (PMC8223752; doi:10.3389/fimmu.2021.638066)
Supplement: Supplementary file 9 [file Presentation_1.pdf]

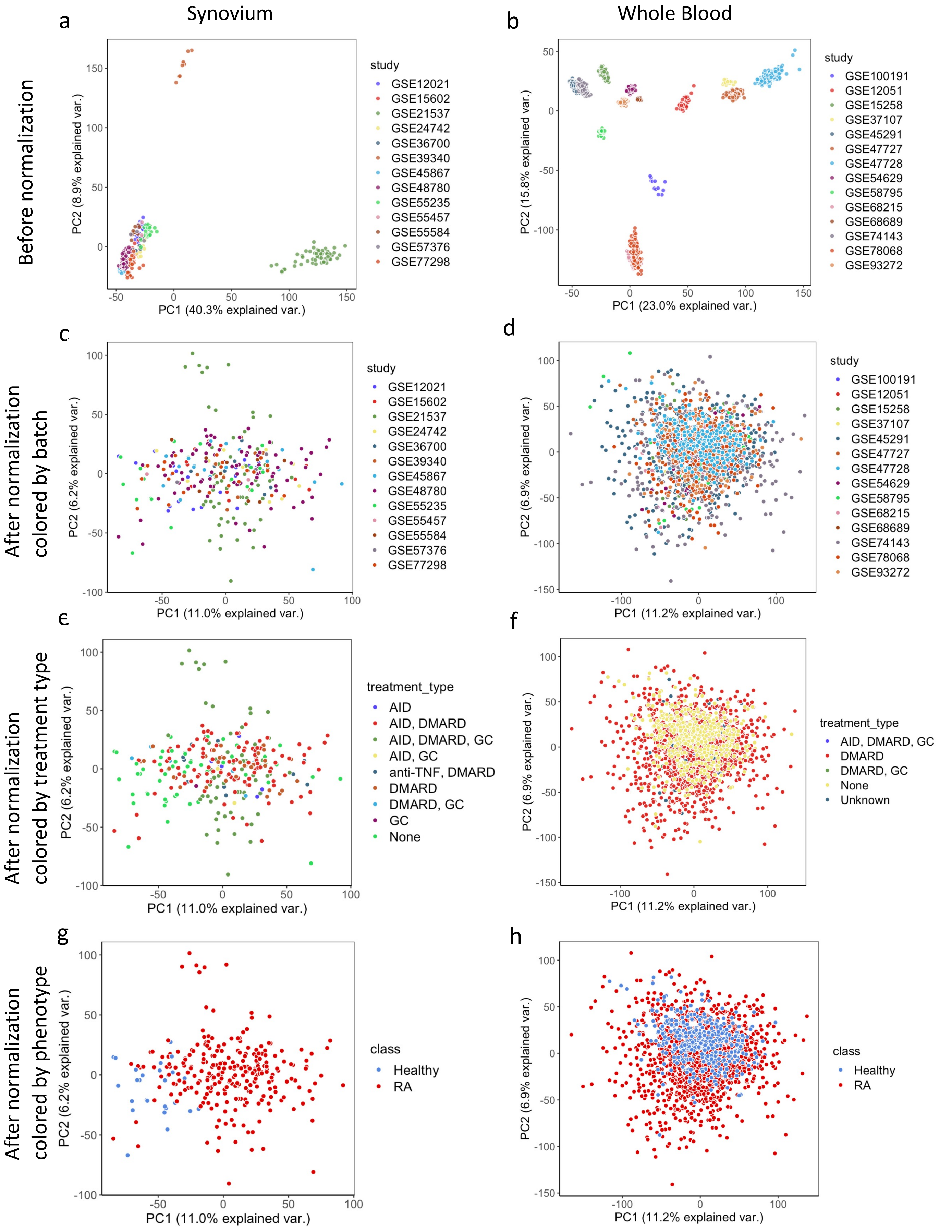

Synovium
Whole Blood
a
b
c
d
e
f
g
h
Before normalization
After normalization colored by batch
After normalization colored by treatment type
After normalization colored by phenotype
PC1 (40.3% explained var.)
PC2 (8.9% explained var.)
PC1 (23.0% explained var.)
PC2 (15.8% explained var.)
PC1 (11.0% explained var.)
PC2 (6.2% explained var.)
PC1 (11.2% explained var.)
PC2 (6.9% explained var.)
study
GSE12021
GSE15602
GSE21537
GSE24742
GSE36700
GSE39340
GSE45867
GSE48780
GSE55235
GSE55457
GSE55584
GSE57376
GSE77298
study
GSE100191
GSE12051
GSE15258
GSE37107
GSE45291
GSE47727
GSE47728
GSE54629
GSE58795
GSE68215
GSE68689
GSE74143
GSE78068
GSE93272
treatment_type
AID
AID, DMARD
AID, DMARD, GC
AID, GC
anti-TNF, DMARD
DMARD
DMARD, GC
GC
None
treatment_type
AID, DMARD, GC
DMARD
DMARD, GC
None
Unknown
class
Healthy
RA

i

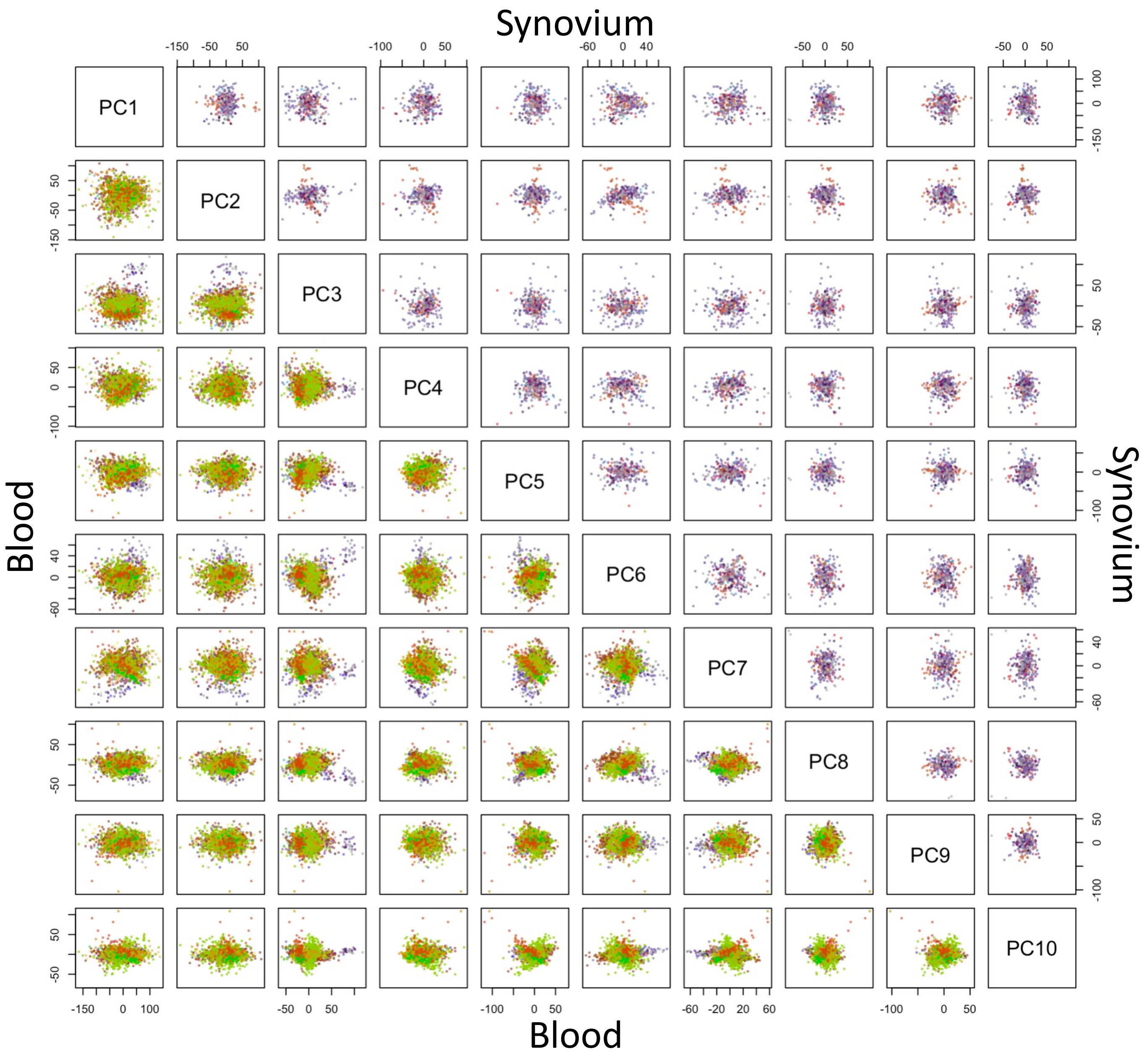

j

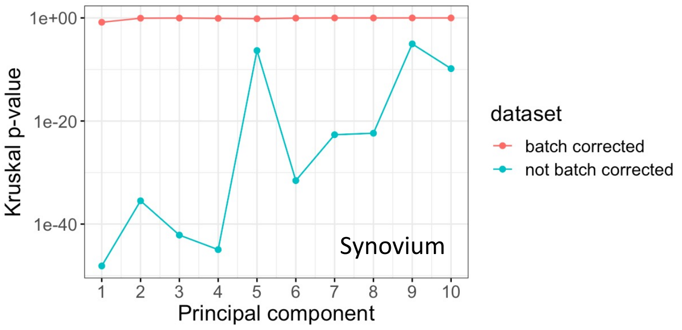

k

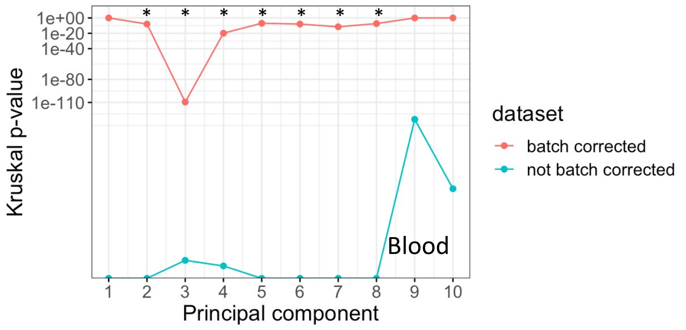

Supplementary Figure 1. Discovery Data Batch Correction, QC and PCA plots

For synovium and whole blood: a) and b) before batch correction; c) and d) after normalization colored by batch; e) and f) after normalization colored by treatment type; g) and h) after normalization colored by phenotype. i) Scatter plot of the first 10 principal components of the batch corrected synovium (upper panel) and blood (lower panel) datasets.

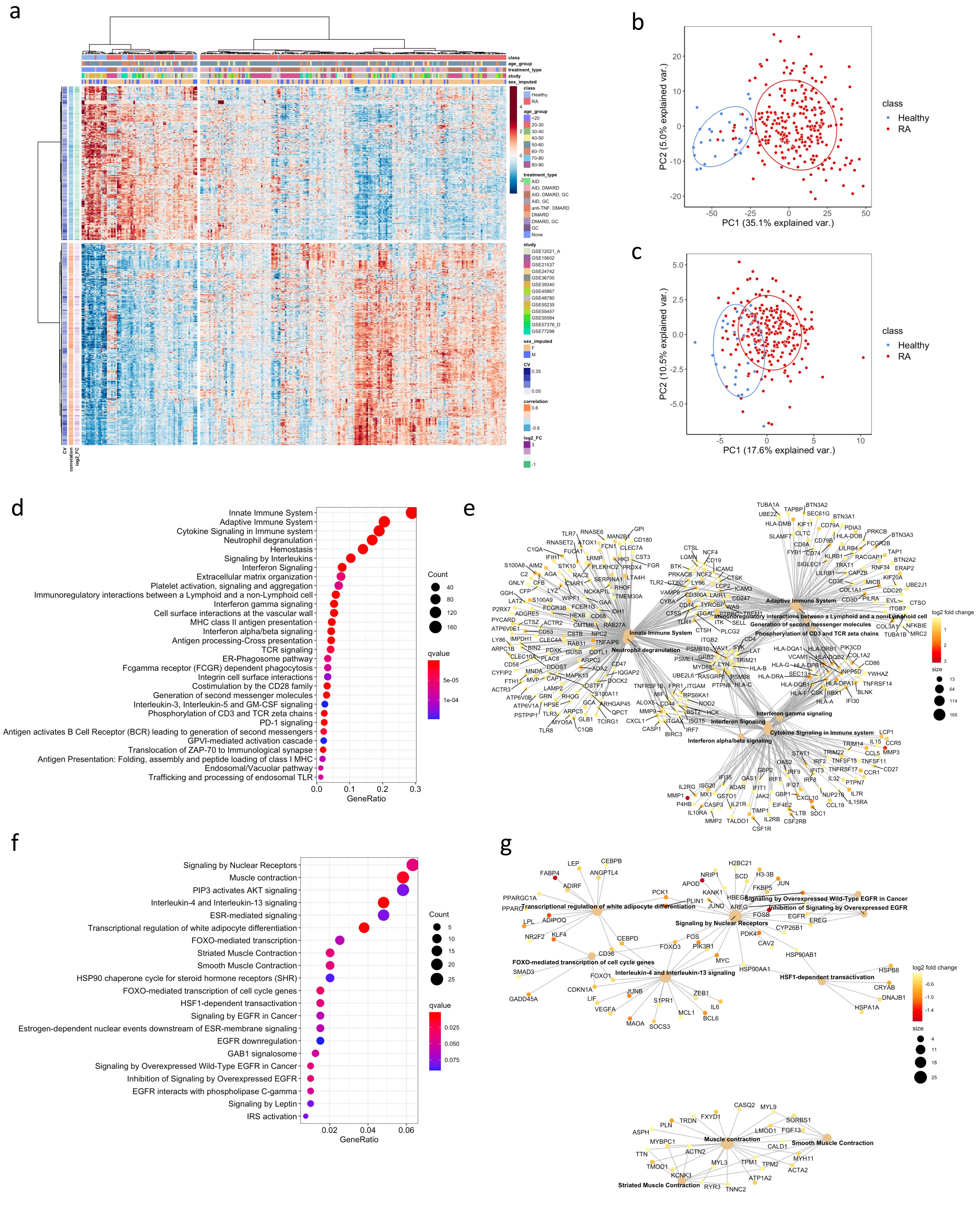

Supplementary Figure 2. Synovium Differential Expression and Pathway Analysis

DGE analysis in synovial tissue. a) Heatmap and b) PCA plot with DE genes. c) PCA plot with the 33 random genes. Reactome pathways for d) , e) up- and f), g) down-regulated genes.

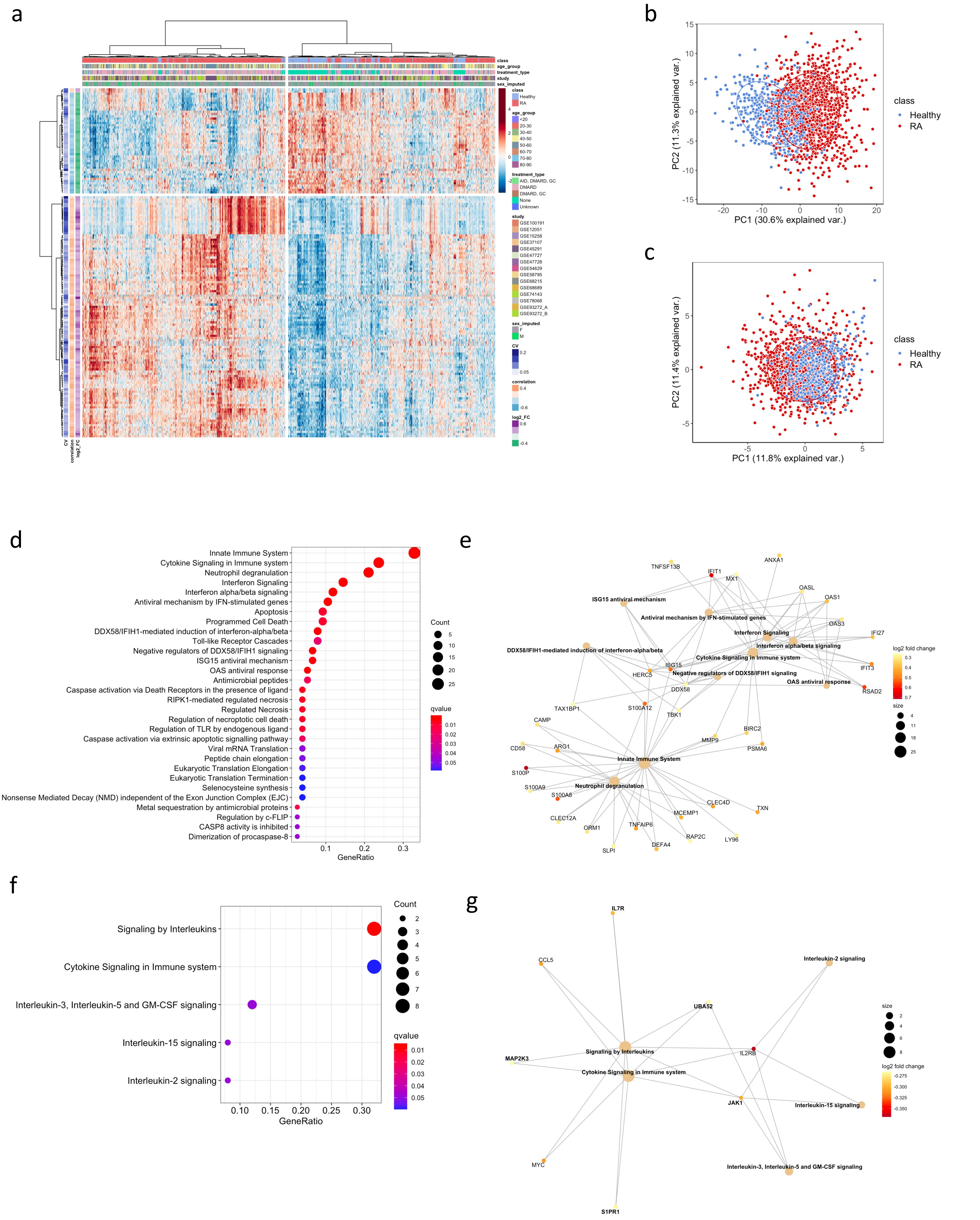

Supplementary Figure 3. Synovium Differential Expression and Pathway Analysis

DGE analysis in whole blood. a) Heatmap and b) PCA plot with DE genes. c) PCA plot with the 33 random genes. Reactome pathways for d) , e) up- and f), g) down-regulated genes.

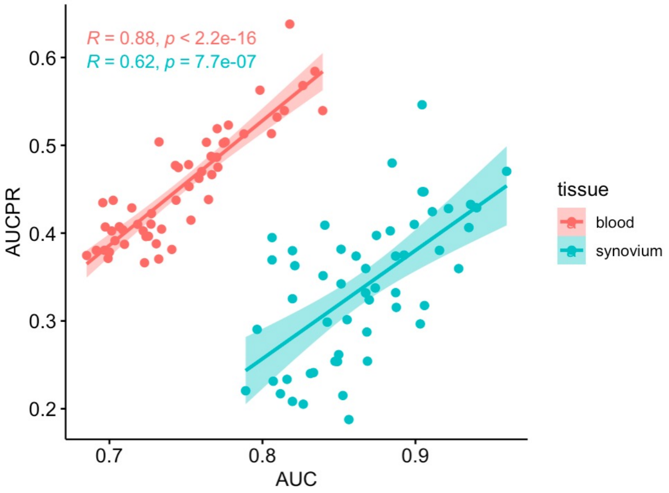

Supplementary Figure 4. Pearson correlation between AUROC and AUCPR for testing synovium and blood datasets.

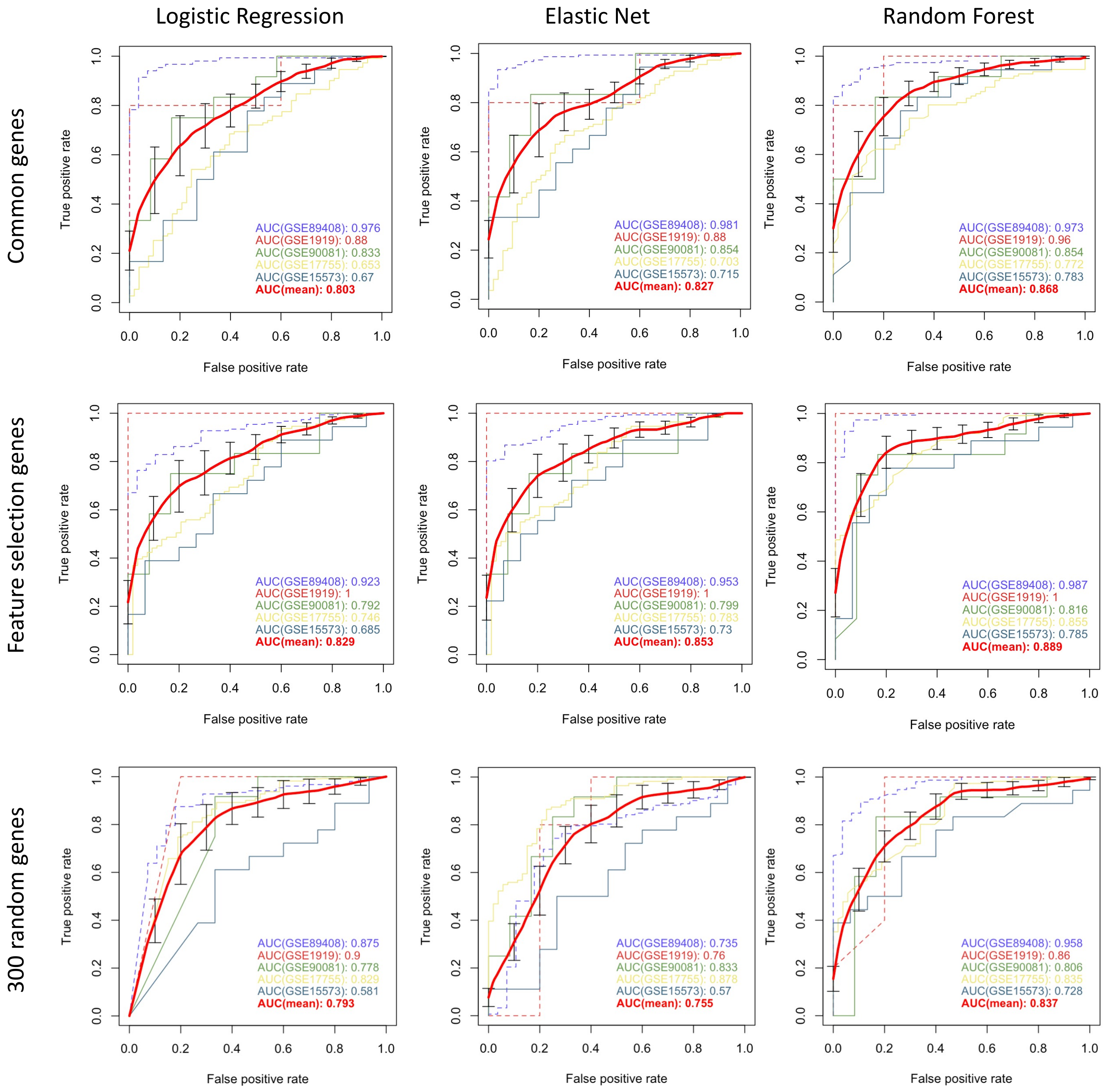

Supplementary Figure 5. Machine Learning Model Comparison

AUROC plots for common and feature selected genes. Three models, a Logistic Regression, Elastic Net and Random Forest, were trained on the discovery whole blood data using either common genes, feature selected genes and 300 random genes and validated on 5 validation datasets. The summary curves are the averaged curves with bars of standard errors and colored by red. The dashed and solid lines represent synovium and blood data, respectively.

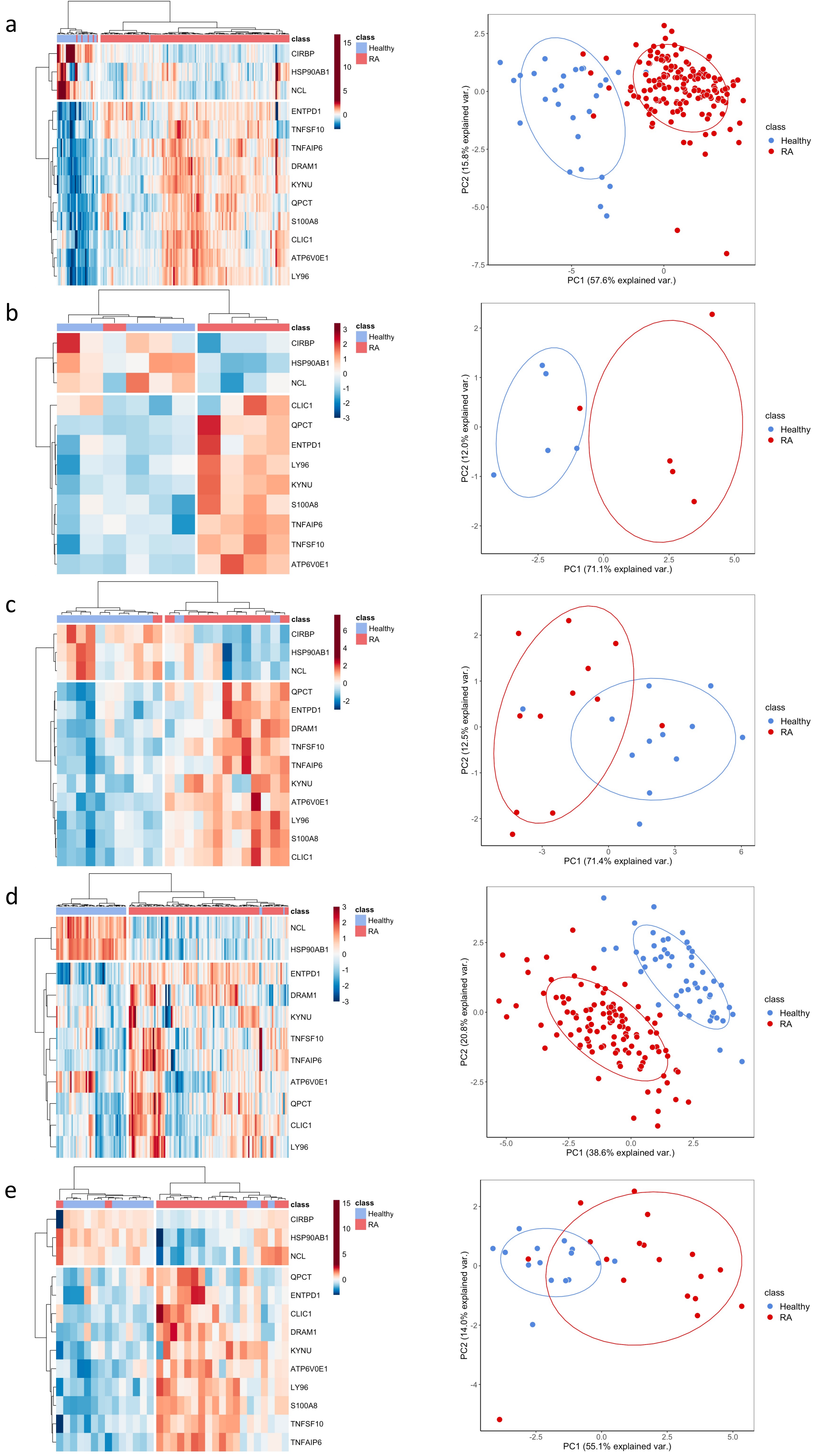

Supplementary Figure 6. Validation Data Batch Correction, QC and PCA Plots

Heatmap and PCA plots of 13 best performing genes on the independent validation a) synovium RNA-seq GSE89408, b) synovium microarray GSE1919, c) whole blood microarray GSE90081, d) PBMC RNA-seq GSE17755 and e) PBMC microarray GSE15573 datasets.

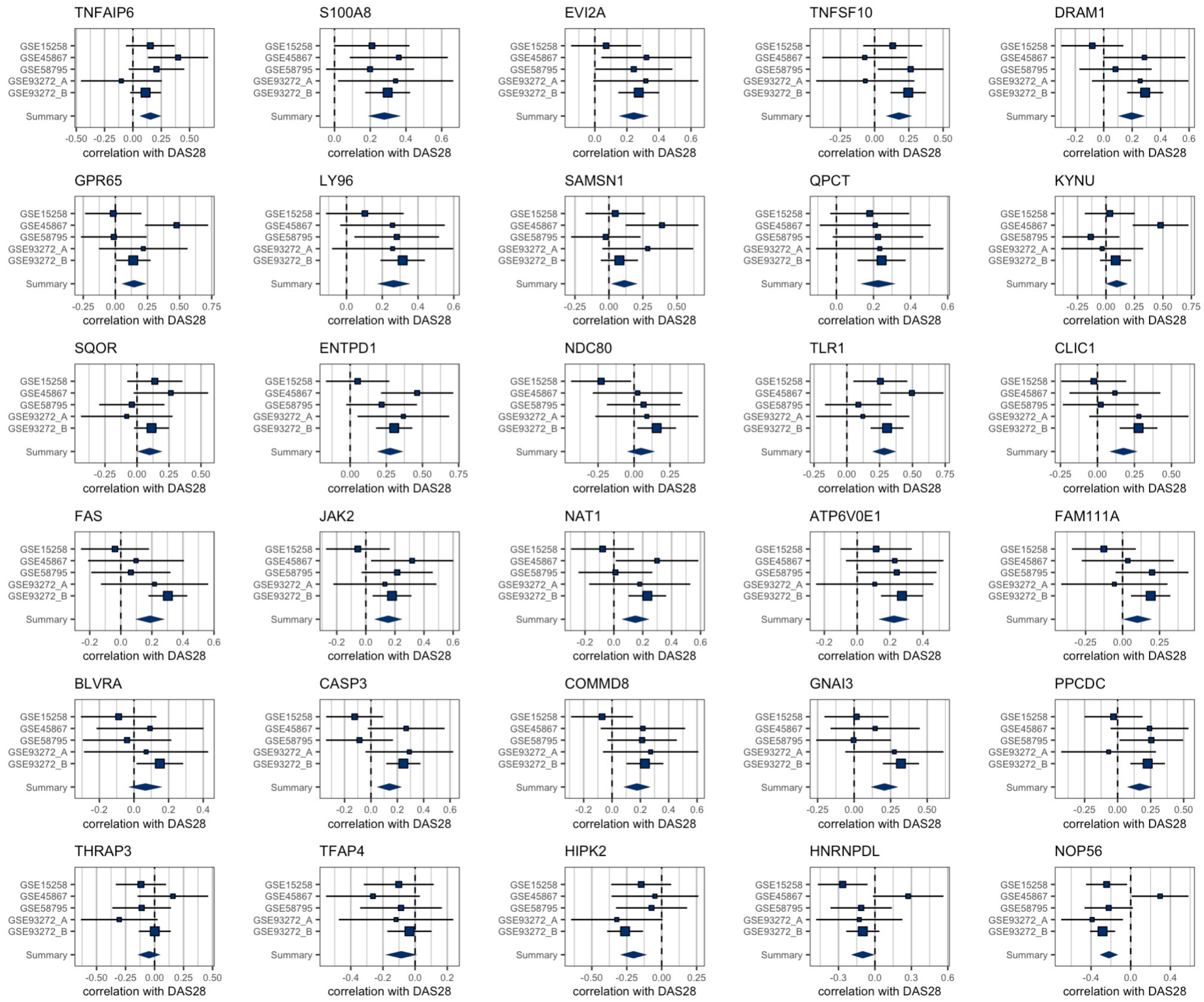
TNFAIP6
S100A8
EVI2A
TNFSF10
DRAM1
GPR65
LY96
SAMSN1
QPCT
KYNU
SQOR
ENTPD1
NDC80
TLR1
CLIC1
FAS
JAK2
NAT1
ATP6V0E1
FAM111A
BLVRA
CASP3
COMMD8
GNAI3
PPCDC
THRAP3
TFAP4
HIPK2
HNRNPDL
NOP56
GSE15258
GSE45867
GSE58795
GSE93272_A
GSE93272_B
Summary
correlation with DAS28

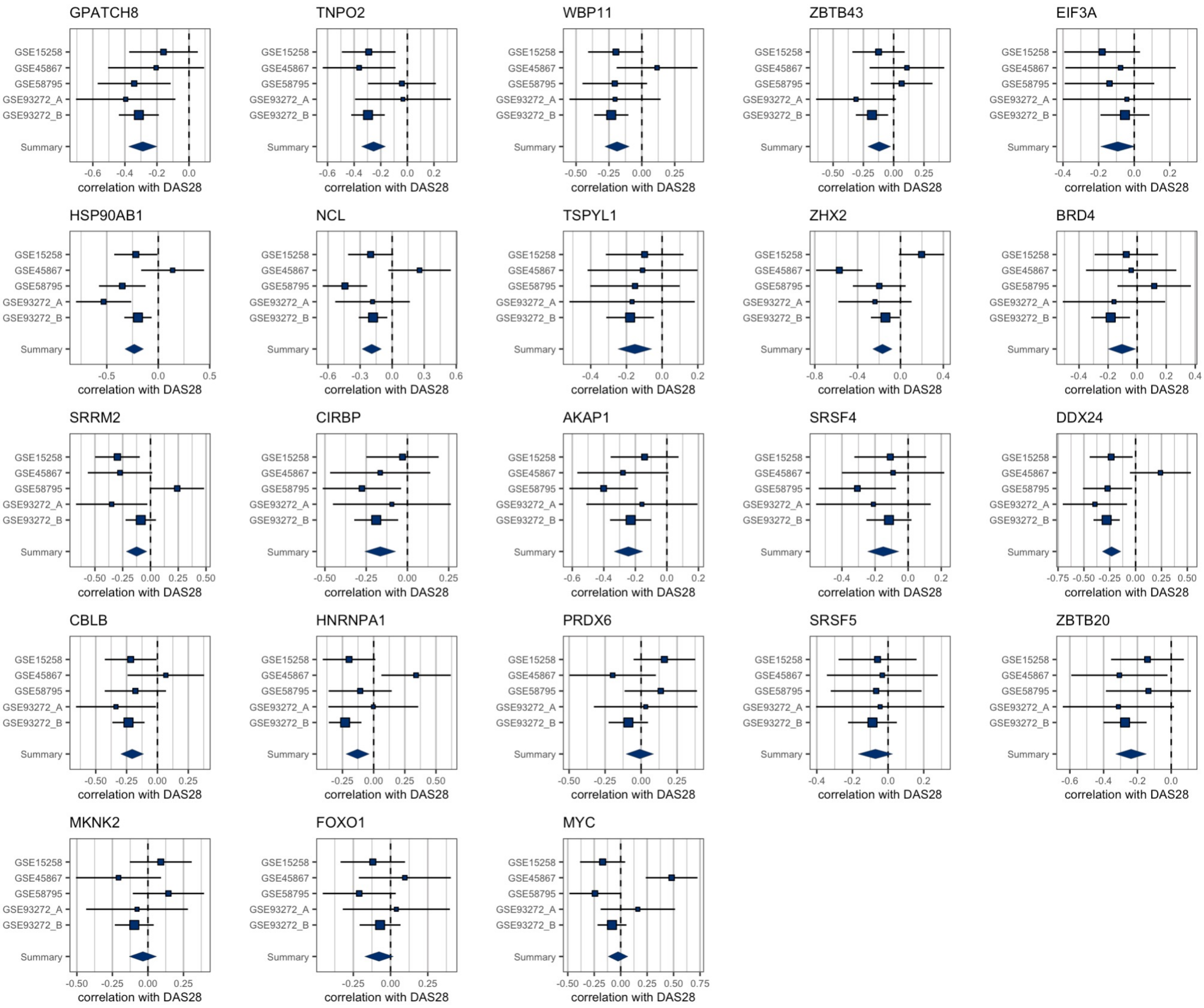

Supplementary Figure 7. Correlation forest plots with DAS28 for all 53 feature selected genes.

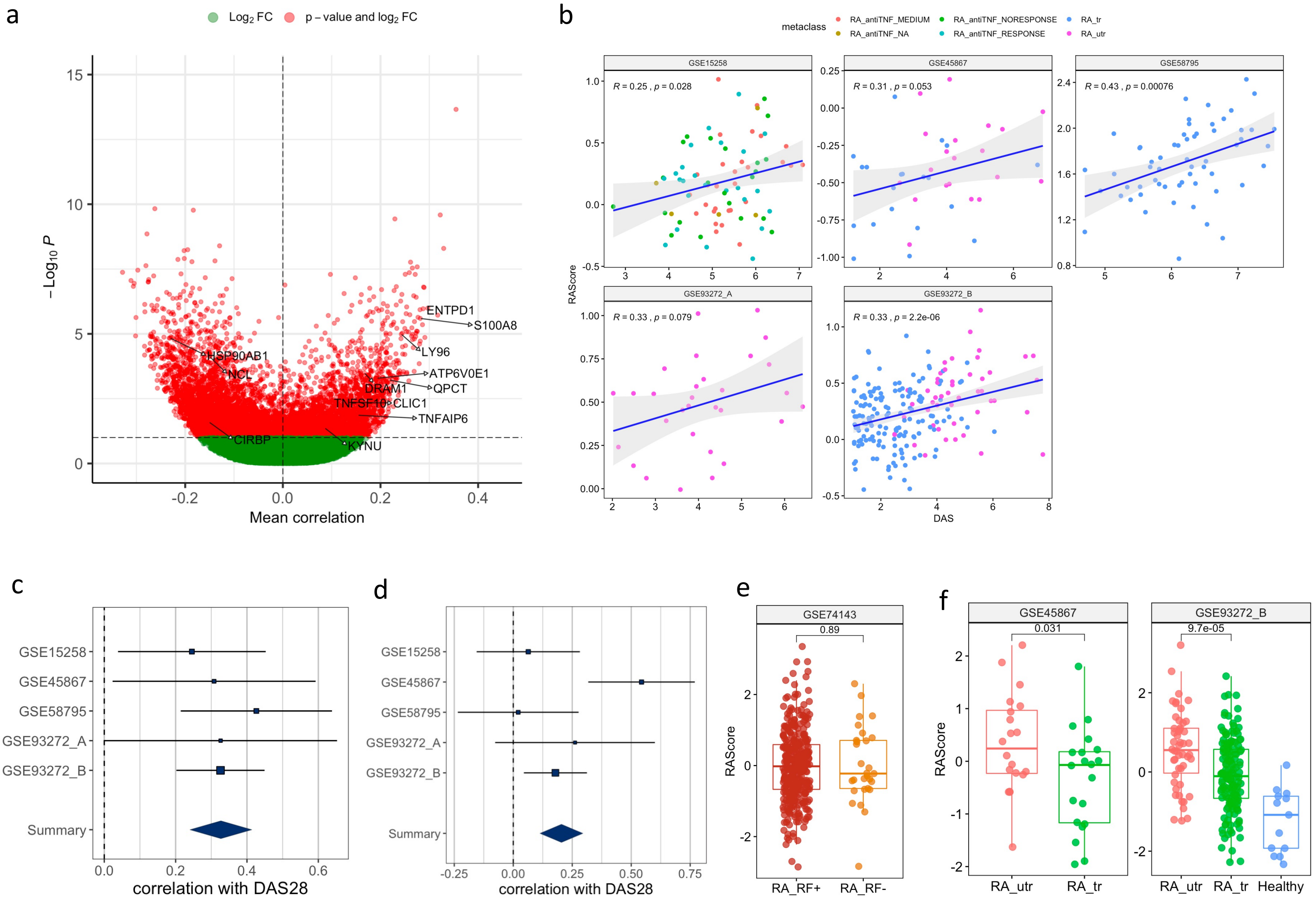

Supplementary Figure 8. The RA Score correlates with DAS28 score, might track the treatment effect and independent of Rheumatoid Factor. a) Volcano plot of correlations of DAS28 with all genes. The RAScore panel genes are highlighted. b) The correlation of the RA Score with DAS28 in synovium GSE45867 and blood GSE15258, GSE58795, GSE93272 datasets. A summary as a forest plot of correlation of DAS28 with c) a score from 13 random genes and d) RAScore. e) The RA Score shows no difference between RF+ and RF- phenotypes. f) The RA Score shows the possible treatment effect in both synovium and blood. RA_utr: RA untreated. RA_tr: RA treated.

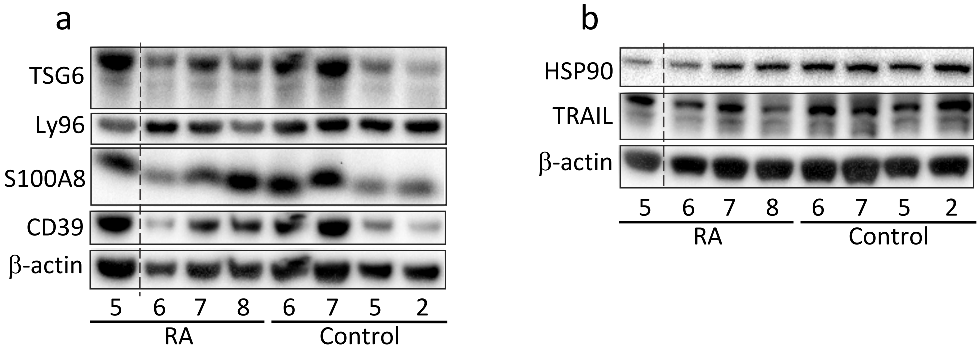

Supplementary Figure 9. The second set of western blot gels. a) TSG6, Ly96, S100A8, CD39 and b) HSP90, TRAIL proteins.
